# Immune cell-lipoprotein imbalance as a marker for early diagnosis of non-small cell lung cancer metastasis

**DOI:** 10.3389/fonc.2022.942964

**Published:** 2022-10-24

**Authors:** Wei Zhang, Weiwei Wang, Junlu Wu, Jiale Tian, Wenhui Yan, Yi Yuan, Yiwen Yao, Anquan Shang, Wenqiang Quan

**Affiliations:** ^1^ Department of Laboratory Medicine, Shanghai Tongji Hospital, School of Medicine, Tongji University, Shanghai, China; ^2^ Department of Pathology, Tinghu People’s Hospital, Yancheng, China; ^3^ Department of Laboratory Medicine, Yangzhi Rehabilitation Hospital (Shanghai Sunshine Rehabilitation Center), Tongji Univeirsity School of Medicine, Shanghai, China; ^4^ Department of Internal Medicine V-Pulmonology, Allergology, Respiratory Intensive Care Medicine, Saarland University Hospital, Homburg, Germany

**Keywords:** NLR, LMR, HNR, NSCLC, risk assessment, diagnostic markers

## Abstract

The underlying molecular mechanisms and evolutionary patterns of lung cancer metastasis remain unclear, resulting in a lack of effective indicators for early diagnosis of metastasis. We retrospectively analyzed 117 patients with primary non-small cell lung cancer (NSCLC) admitted to Tongji Hospital of Tongji University in 2021, of which 93 patients with tumor metastasis were set as the metastasis group. 24 patients without metastasis were set as the non-metastasis group. The differences of each index in the two groups of patients and the expression levels in different TNM stages were compared. This study intends to evaluate the diagnostic value and net clinical benefit of common blood-related indicators Neutrophil/lymphocyte (NLR), lymphocyte/monocyte (LMR), High density lipoprotein/neutrophil (HNR), High density lipoprotein/monocyte (HMR) and combined assays in NSCLC metastasis for the early diagnosis of patients with NSCLC metastasis. It was found that the level of NLR was higher in metastatic NSCLC than non-metastatic, but the level of LMR, HNR and HMR was lower. The levels of NLR, LMR, HNR and HMR in patients with different TNM stages showed that NLR levels increased with TNM stage, while LMR, HNR and HMR levels decreased. The threshold probability range of the 4 combined tests was greater and the overall clinical benefit rate was higher compared to the individual tests. Our findings suggest that NLR, LMR, HNR and HMR have better diagnostic value for NSCLC metastasis. This study provides a clinical basis for investigating the mechanisms by which immune cells and lipid metabolism-related proteins remodel the microenvironment prior to NSCLC metastasis.

## Introduction

Lung cancer is the second most common cancer in the world in terms of human incidence, is highly aggressive and metastatic, and is the leading cause of cancer deaths (1, 2). Studies show (3),that 2.2 million people in the United States were diagnosed with lung cancer in 2018 alone, and nearly 1.6 million died from lung cancer. Non-small cell lung cancer (NSCLC) accounts for 85% of lung cancers and is the common type of lung cancer, including squamous, adenocarcinoma and large cell lung cancer (4–6). Reports show that patients with NSCLC generally have poor treatment outcomes, with an overall 5-year survival rate of less than 15% (7). The main reason for this is that patients develop asymptomatic cancer metastases, which increases the risk of treatment and mortality for patients (8), and the 5-year survival rate for lung cancer patients without metastatic spread is about 35%, however, the 5-year survival rate for lung cancer patients with metastases is less than 5% (9), and patient death due to metastases However, the 5-year survival rate of lung cancer patients with metastasis is less than 5% (10), and death due to metastasis is the main cause of death. Therefore, the occurrence of early metastasis in lung cancer patients is of great importance for the selection of appropriate treatment plans. Currently, non-invasive tests such as long-non-coding RNA (Lnc RNA), neuron-specific enolase (NSE), circulating tumor DNA (ct DNA) and microRNAs have good diagnostic value for early diagnosis and cancer staging of lung cancer (11–14), however, there are few reports on the diagnosis of metastasis in non-small cell lung cancer. Liquid biopsy and tissue biopsy are the gold standard for cancer diagnosis, however, due to the limitations of their operation such as invasiveness, complexity, inability to monitor longitudinally and continuous monitoring, and clinical variability of patients (15, 16), monitoring cannot be routinely performed in lung cancer patients. There are still no non-invasive markers that effectively predict the development of metastasis in patients with non-small cell lung cancer.

Inflammation is one of the markers of the body’s immune status and also an important factor affecting the tumor microenvironment, which can play a role in the occurrence and development of various types of cancer by promoting cancer cell proliferation and metastasis (17).Inflammation plays a very important role in carcinogenesis and development, and chronic inflammation accompanies all stages of tumorigenesis. Complete blood counts such as neutrophils, single core packs, and lymphocytes are routine preoperative tests used to reflect the inflammatory status of the body, and reports show (18, 19)that these indicators have good diagnostic performance in the early diagnosis and prognosis of many tumors such as colorectal cancer and nasopharyngeal carcinoma. Many cancers lead to metabolic disorders in the body, and disorders of lipid metabolism have been reported to play a key role in the pathogenesis of cancer. Studies have found correlations between serum lipids and many types of tumors such as breast cancer and gastric cancer (20). High-density lipoprotein cholesterol, a serum lipid, has been shown to correlate with tumor incidence and mortality (21). Neutrophil/lymphocyte (NLR), lymphocyte/monocyte (LMR), HDL cholesterol/neutrophil (HNR), and HDL cholesterol/monocyte (HMR) are calculated from blood counts and parameters in serum lipids, which have also been shown to be predictive of tumor prognosis (22). This study was designed to evaluate the diagnostic value of NLR, LMR, HNR, HMR and the combination of the four in patients with NSCLC metastases, to determine the optimal threshold values, and to assess the net clinical benefit of each individual and combined test for the early diagnosis of patients with NSCLC metastases.

## Materials and methods

### Medical record information

Retrospective analysis of 117 patients first diagnosed with NSCLC admitted to Tongji Hospital of Tongji University in 2021, the diagnostic staging criteria of non-small cell lung cancer were based on: the International Association for the Study of Lung Cancer (IASLC) published the eighth edition of TNM staging of lung cancer (23, 24), according to the TNM staging criteria, T stage T0 *in situ* tumor, T1-T2 stage tumor encircling lung tissue and dirty layer pleura, tumor invasion of The 24 patients who met all the criteria were regarded as non-metastatic and set up as the non-metastatic group, including T0 stage *in situ* tumor, T1-T2 stage tumor encircling lung tissue and dirty pleura, tumor invading the main bronchus, no regional lymph node metastasis in N stage, and no distant metastasis in M stage. In stage T2, the tumor invaded the main bronchus, invaded the dirty pleura, invaded any organ in stage T3-T4; in stage N1, ipsilateral hilar lymph node and intrapulmonary lymph node metastasis, and in stage N2-N3; in stage M, the tumor met M1 by distant metastasis, and 93 patients who met any of the above mentioned criteria or above were considered to have metastasis and set up as the metastasis group.

Patients with NSCLC are examined by one or more types of imaging, such as chest radiography, CT, magnetic resonance imaging (MRI), positron emission tomography-computed tomography (PET-CT), ultrasound, etc. Imaging, histological specimens by ultrasound or CT-guided percutaneous lung biopsy, superficial lymph node or subcutaneous node biopsy, pleurodesis biopsy or thoracoscopic pleural biopsy, bronchoscopy and sampling biopsy, and one or more histological or cytological examinations.

### Inclusion and exclusion criteria

Inclusion criteria: patients with primary NSCLC diagnosed by surgical pathology, not treated with radiotherapy before enrollment, not receiving radiotherapy and other antitumor treatments, no recent infectious symptoms and chronic infectious diseases.

Exclusion criteria: those with other types of tumors, those with missing clinical data, those with uncertain clinical stage or metastasis, those with organ dysfunction, those with hematologic disorders, those with immune deficiencies, and those with autoimmune diseases.

### Clinical information and patient examination

Patients’ gender, age, smoking history, neutrophils (ANC), lymphocytes (ALC), monocytes (AMC), platelets (PLT), glucose (GLU), triglycerides (TG), cholesterol (TC), high-density lipoprotein (HDL), low-density lipoprotein (LDL), TNM stage, and type of lung cancer were included in the monitoring indexes, and the inclusion of monitoring indexes was based on the patient’s visit to treatment Blood samples were collected for the first time prior to treatment, and neutrophils, lymphocytes, monocytes, and platelets were measured using a Myriad BC-6800 machine, and blood glucose, triglycerides, cholesterol, HDL, and LDL were measured using a Beckman AU5800 machine. Because the data were analyzed retrospectively, written informed consent from the subjects was waived.

### Statistical analysis

SPSS 25.0 was used to statistically analyze the data that met the requirements, and the normal test was used to analyze the measurement data according to the shapiro-wilk test, and the normally distributed data were expressed by (`x ± SD), and the t-test for two independent samples was used to compare between groups of normally distributed measurement data, and the c2 test or fisher’s exact probability method. The median (M) and percentile (P25, P75) were used to express the measures of skewed distribution, and the Mann-Whitney U test was used to compare the measures of skewed distribution between two groups, and the Kruskal-Wakkis H test for independent samples was used to compare the measures of skewed distribution between multiple groups, and pairwise comparisons between groups were performed for the overall test with differences, and pairwise comparisons between groups were performed using the The Bonferroni method was used to correct for significance levels. The ROC curves of the relevant indexes and combined tests were plotted using graphpad prism software, and the sensitivity, specificity, best critical value, Youden index, negative predictive value (NPV) and positive predictive value (PPV) of each relevant index in NSCLC metastasis were calculated using medcalc software, and the accuracy of the test was judged by the area under the curve (AUC). Binary logistic regression analysis was used to calculate the joint predictors of NLR, LMR, HNR, and HMR, and Delong test test was used to compare the AUC of each index. The cutoff values and quartiles (P25, P50, P75) were used as cutoff points, respectively, and the risk of NLR, LMR, HNR, and HMR levels in NSCLC metastasis was evaluated using binary logistic regression analysis. Clinical decision curves (DCA) were drawn using R software 4.1.0 (https://www.r-project.org/) to evaluate the net clinical benefit of NLR, LMR, HNR, HMR and the 4 joint trials. The net clinical benefit rate for each index was determined by the net benefit at different threshold probabilities, which was calculated by subtracting the proportion of false-positive patients from the proportion of true-positive patients and by weighing the relative harms of forgoing the intervention against the negative consequences of unnecessary intervention. This study used the rmda package in R software 4.1.0. Differences were considered statistically significant at P < 0.05.

## Results

### Comparison of clinical baseline information between the two groups of patients

The differences were not statistically significant (P>0.05) when comparing gender, age, tumor type, smoking history, PLT, ANC, GLU, TG, TC, HDL and LDL between the two groups, and the differences were statistically significant (P<0.05) when comparing TNM stage, ALC and AMC between the two groups, (**Table 1**).

**Table 1:** The analysis of the clinical data of the NSCLC patients in the metastatic and nonmetastatic groups.

Inspection items		No transfer group (n=24)	Transfer group (n=93)	c2/t/z value	P value
**Gender**	**male**	17	77	1.054	0.305
	**female**	7	16		
**Age (year)**		62.5 (53, 71)	66 (60,71)	1.301	0.193
**History of smoking**	**no**	8	23	0.725	0.395
	**Yes**	16	70		
**Type**	**LACC**	12	39	–	0.149
	**SQCC**	10	52		
	**LCLC**	2	2		
**TNM**	**I**	9	0	–	<0.001
	**II**	15	2		
	**III**	0	29		
	**IV**	0	62		
**ANC (×109/L)**		3.25 (2.35,4.10)	3.70 (2.55,4.90)	-1.486	0.137
**ALC (×109/L)**		1.40 (0.90,1.80)	1.00 (0.70,1.30)	-2.884	0.004
**AMC (×109/L)**		0.40 (0.23,0.50)	0.50 (0.40,0.65)	-2.324	0.020
**PLT (×109/L)**		175 (137,234)	192 (134,239)	-0.614	0.539
**GLU (mmol/L)**		4.67 (4.20,5.15)	4.79 (4.37,5.72)	-1.144	0.253
**TG (mmol/L)**		1.19 (0.75,1.55)	1.02 (0.81,1.53)	-0.240	0.811
**TC (mmol/L)**		4.61 ± 1.27	4.27 ± 0.97	-1.114	0.265
**HDL (mg/dl)**		44.00 (39.58,52.76)	39.63 (32.46,48.08)	-1.856	0.063
**LDL (mg/dl)**		106.14 (75.34,138.37)	97.10 (79.90,115.89)	-0.749	0.454

### Expression levels of NLR, LMR, HNR, HMR in two groups of patients

**Figure 1:**
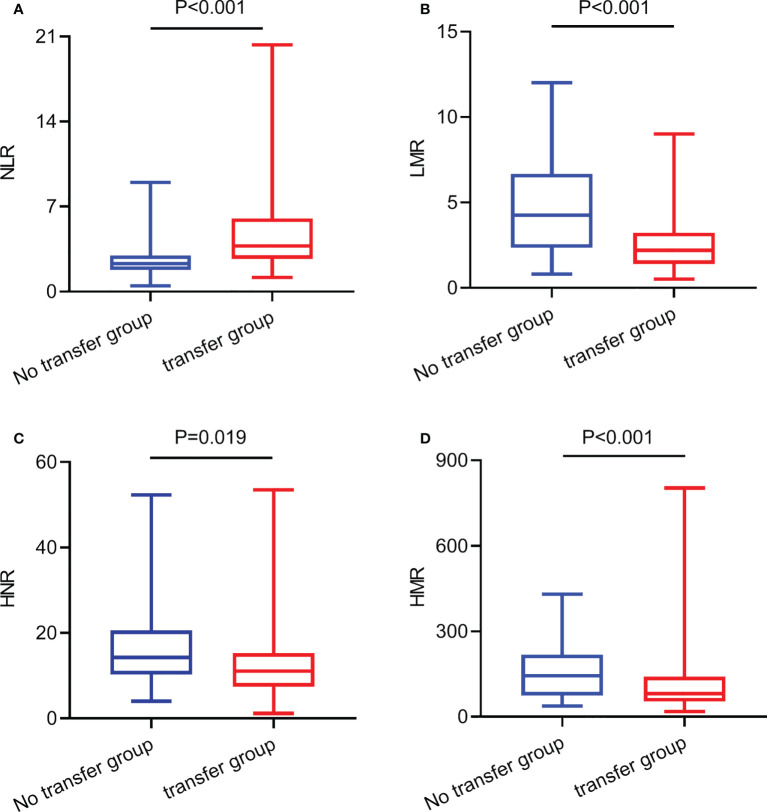
The expression levels of NLR, LMR, HNR, and HMR in metastatic and non-metastatic NSCLC patients. **(A)** The expression levels of NLR in metastatic and non-metastatic NSCLC patients. **(B)** The expression levels of LMR in metastatic and non-metastatic NSCLC patients. **(C)** The expression levels of HNR in metastatic and non-metastatic NSCLC patients. **(D)** The expression levels of HMR in metastatic and non-metastatic NSCLC patients.

The level of NLR in the lung cancer metastasis group was higher than that in the non-transferred group, and the difference was statistically significant (Z=-3.584, P<0.001), (**Figure 1A**); the level of LMR in the lung cancer metastasis group was lower than that in the non-transferred group, and the difference was statistically significant (Z=-3.691, P<0.001), (**Figure 1B**); the level of HNR in the lung cancer metastasis group was lower than that in the non-transferred group, and the difference was statistically significant (Z=-2.352, P= 0.019), (**Figure 1C**); the HMR level in the lung cancer metastasis group was lower than that in the non-metastatic group, and the difference was statistically significant (Z=-2.518, P<0.001), (**Figure 1D**).

### Expression levels of NLR, LMR, HNR, and HMR in patients with different stages

**Figure 2:**
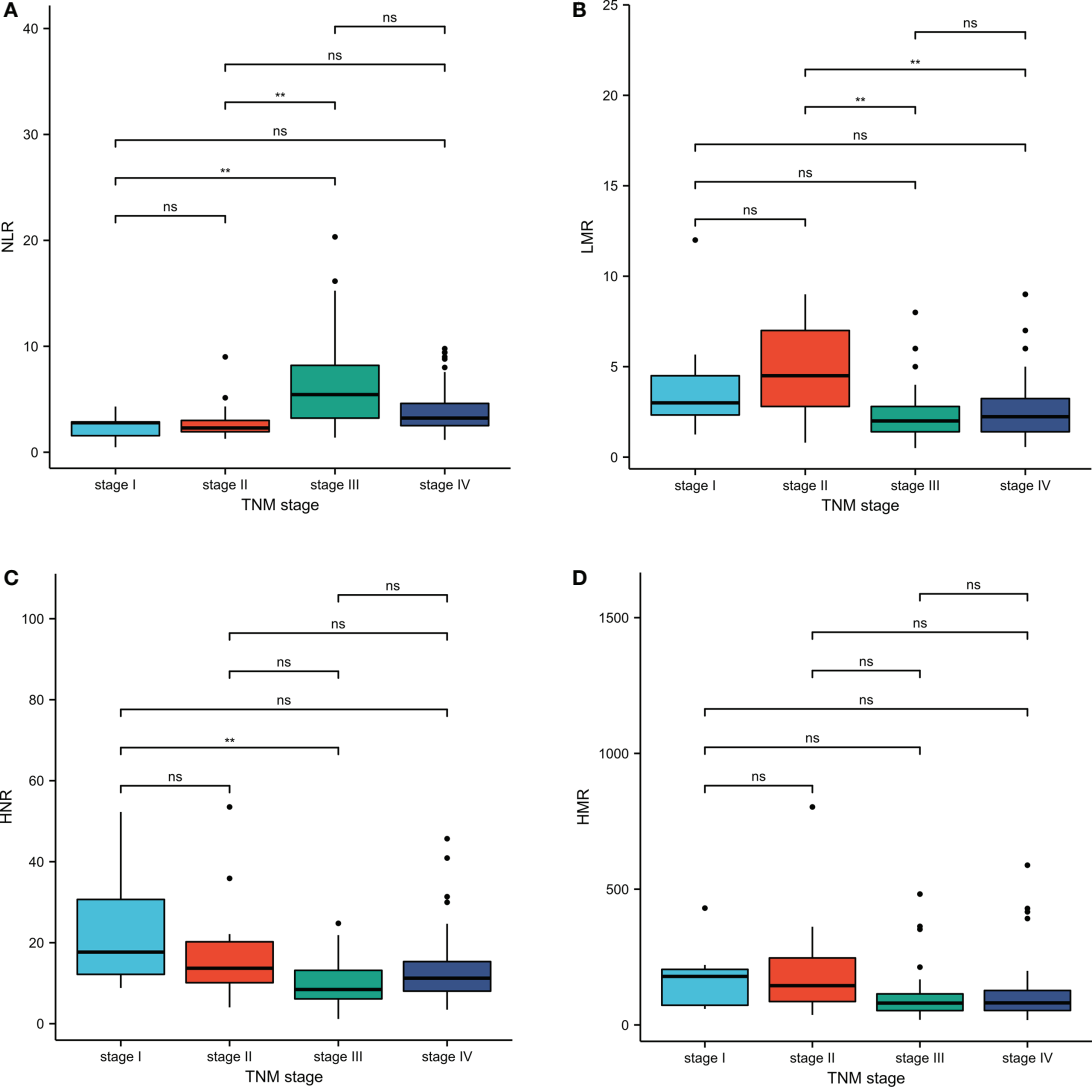
Inter-group comparison of NLR, LMR, HNR, and HMR in different stages of metastatic and non-metastatic NSCLC groups. **(A)** Inter-group comparison of NLR in different stages of metastatic and non-metastatic NSCLC groups. **(B)** Inter-group comparison of LMR in different stages of metastatic and non-metastatic NSCLC groups. **(C)** Inter-group comparison of HNR in different stages of metastatic and non-metastatic NSCLC groups. **(D)** Inter-group comparison of HMR in different stages of metastatic and non-metastatic NSCLC groups. ns, p≥0.05; **p<0.01.

TNM stage III patients had higher NLR levels than stage I and stage II, with statistically significant differences (P < 0.01); stage III and stage IV patients had lower LMR levels than stage II, with statistically significant differences (P < 0.01); stage III patients had lower HNR levels than stage I, with statistically significant differences (P<0.01); stage III patients had lower HMR levels than stage I, with statistically significant differences (P<0.01). The difference was statistically significant (P<0.01); the difference was not statistically significant (P>0.05) when comparing the HMR levels of patients in each stage of TNM (**Table 2**; **Figure 2**).

**Table 2:** The expression levels of NLR, LMR, HNR, and HMR in patients of different stages.

Group	Number	NLR	LMR	HNR	HMR
stage I	9	2.78 (1.56,2.86)	3.00 (2.33,4.50)	17.68 (12.18,30.68)	178.60 (72.40,204.53)
stage II	17	2.29 (1.93,3.00)	4.50 (2.80,7.00)	13.69 (10.12,20.23)	144.58 (86.10,246.70)
stage III	29	5.44 (3.22,8.20)	2.00 (1.40,2.80)	8.44 (6.13,13.17)	80.44 (52.78,114.33)
stage IV	62	3.23 (2.51,4.61)	2.24 (1.40,3.24)	11.24 (8.04,15.34)	81.27 (53.12,126.85)

### Diagnostic value of each index in NSCLC metastasis

The joint predictors of NLR, LMR, HNR, and HMR were calculated using binary logistic regression analysis with the transferred or untransferred subgroup Y (transferred = 1, untransferred = 0) as the dependent variable and NLR (X1), LMR (X2), HNR (X3), and HMR (X4) as the independent variables, with the regression equation Y = 4.413 + 0.033X1-0.970 X2-0.102X3+0.013X4, and the joint predictors were used as four joint test indicators for outcome analysis.

**Figure 3:**
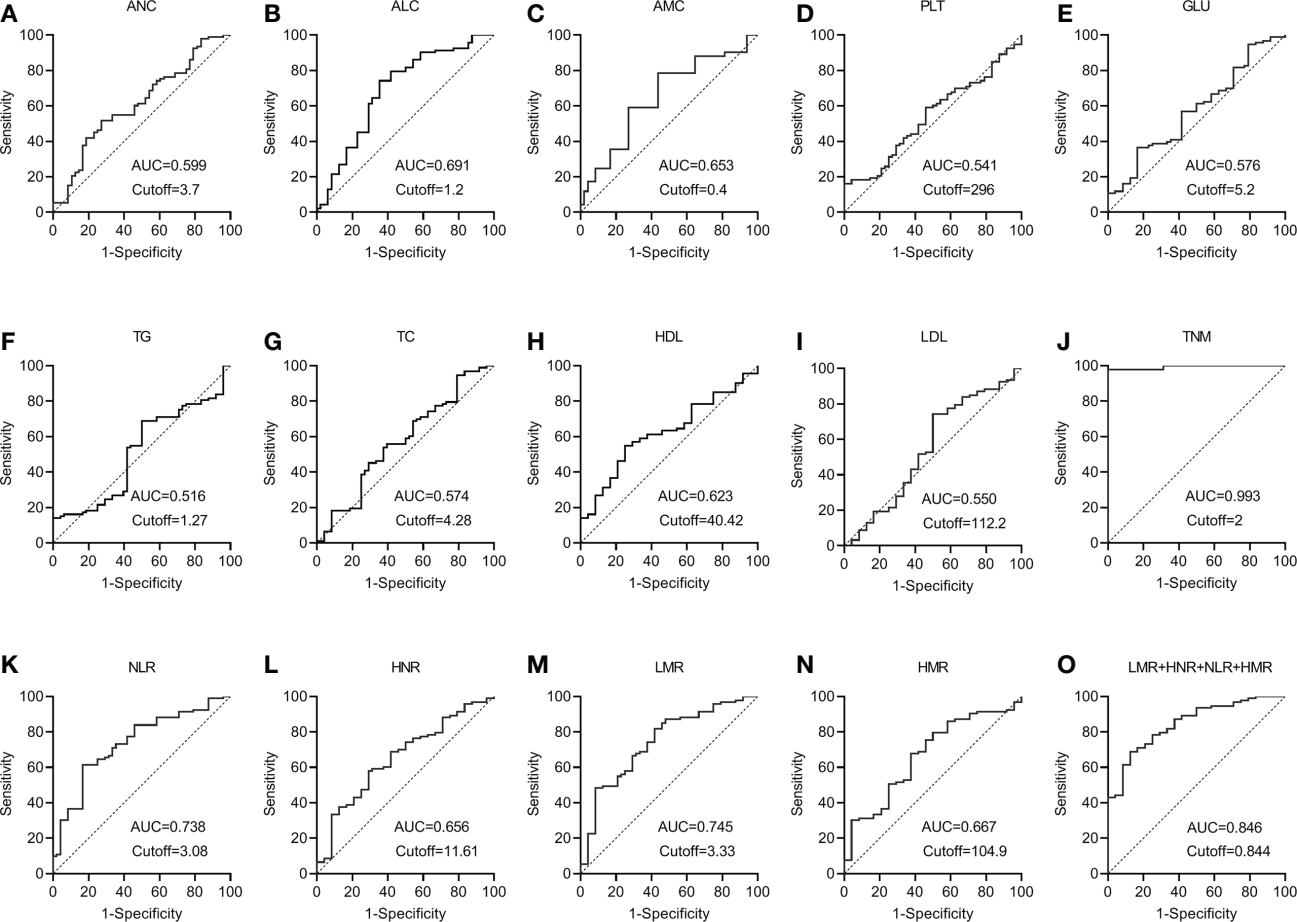
The diagnostic value of different blood markers in NSCLC metastasis **(A)** The diagnostic value of ANC in NSCLC metastasis. **(B)** The diagnostic value of ALC in NSCLC metastasis. **(C)** The diagnostic value of AMC in NSCLC metastasis. **(D)** The diagnostic value of PLT in NSCLC metastasis. **(E)** The diagnostic value of GLU in NSCLC metastasis. **(F)** The diagnostic value of TG in NSCLC metastasis diagnostic value in NSCLC metastasis. **(G)** The diagnostic value of TC in NSCLC metastasis. **(H)** The diagnostic value of HDL in NSCLC metastasis. **(I)** The diagnostic value of LDL in NSCLC metastasis. **(J)** The diagnostic value of TNM in NSCLC metastasis. **(K)** The diagnostic value of NLR in NSCLC metastasis. **(L)** The diagnostic value of HNR in NSCLC metastasis. **(M)** The diagnostic value of LMR in NSCLC metastasis. **(N)** The diagnostic value of HMR in NSCLC metastasis. **(O)** The diagnostic value of LMR+HNR+NLR+HMR in NSCLC metastasis.

ROC curves for each index and combined test were produced using graphpad prism software and are shown in **Figures 3A–O**. The AUC value of the neutrophil assay was 0.599 and the cutoff value was 3.7×10^9^/L, and the sensitivity and specificity were 49.46% and 70.83%, and the PPV and NPV were 86.8% and 26.6%, respectively; the AUC value of the lymphocyte assay was 0.691 and the cutoff value was 1.2×10^9^/L, and the sensitivity and specificity were 74.19% and 62.50%, PPV and NPV were 88.5% and 38.5%, respectively; the AUC value of PLT assay was 0.541 and the cutoff value was 296×10^9^/L, the sensitivity and specificity were 16.13% and 100.00%, PPV and NPV were 100.0% and 23.5%, respectively; the AUC value of GLU assay was The sensitivity and specificity were 36.56%, 83.33%, 89.5% and 25.3% for PPV and NPV, respectively, at an AUC value of 0.576 and a cutoff value of 5.2 mmol/L for GLU; 68.82% and 50.00% for sensitivity and specificity at an AUC value of 0.516 and a cutoff value of 1.27 mmol/L for TG The sensitivity and specificity of the AUC value of 0.574 and the cutoff value of 4.28 mmol/L for TC assay were 53.76% and 62.50%, and the PPV and NPV were 84.7% and 25.9%, respectively; the AUC value of 0.623 and the cutoff value of The sensitivity and specificity were 54.84%, 75.00%, 89.5% and 30.0% for PPV and NPV, respectively, at 40.42 mg/dl; the sensitivity and specificity were 74.19%, 50.00%, and 85.2% and 33.3% for PPV and NPV, respectively, at 0.550 AUC and 112.2 mg/dl for LDL. The sensitivity and specificity of the NLR assay were 61.29% and 83.33% at an AUC value of 0.738 and a cutoff value of 3.08, and the PPV and NPV were 93.4% and 35.7%, respectively; the sensitivity and specificity of the HNR assay were 58.06% and 70.06% at an AUC value of 0.656 and a cutoff value of 11.61, respectively. The sensitivity and specificity of AUC value of 0.656 and cutoff value of 11.61 for HNR were 58.06% and 70.83%, and the PPV and NPV were 88.5% and 30.4%, respectively; the sensitivity and specificity of AUC value of 0.745 and cutoff value of 3.33 for LMR were 81.72% and 58.33%, and the PPV and NPV were 88.4% and 45.2%, respectively; the AUC value of 0.667 and cutoff value of 104.9 The sensitivity and specificity were 67.74%, 62.5%, 87.5% and 33.3% for PPV and NPV, respectively, at an AUC of 0.667 and a cutoff value of 104.9 for HMR; the sensitivity and specificity were 97.85%, 100.00%, and 100.00% for PPV and NPV, respectively, at an AUC of 0.993 and a cutoff value of 2 for TNM staging. 100.00% and 92.3%; the sensitivity and specificity were 68.82% and 87.50%, and the PPV and NPV were 95.5% and 42.0%, respectively, when the AUC value of the 4 joint tests was 0.846 and the cutoff value was 0.844; (**Table 3**).

**Table 3:** The analysis of the expression levels of each test items in NSCLC metastasis.

Inspection items	Youden index	Cutoff value	AUC	AUC 95%CI	Sensitivity	Specificity	PPV (%)	NPV (%)
ANC	0.203	3.7	0.599	0.504-0.688	49.46	70.83	86.8	26.6
ALC	0.367	1.2	0.691	0.599-0.773	74.19	62.50	88.5	38.5
AMC	0.258	0.4	0.653	0.559-0.738	59.14	66.67	87.3	29.6
PLT	0.161	296	0.541	0.446-0.633	16.13	100.00	100	23.5
GLU	0.199	5.2	0.576	0.481-0.667	36.56	83.33	89.5	25.3
TG	0.188	1.27	0.516	0.422-0.609	68.82	50.00	89.5	25.3
TC	0.163	4.28	0.574	0.479-0.665	53.76	62.50	84.7	25.9
HDL	0.298	40.42	0.623	0.529-0.711	54.84	75.00	89.5	30.0
LDL	0.242	112.2	0.550	0.455-0.642	74.19	50.00	85.2	33.3
NLR	0.446	3.08	0.738	0.649-0.815	61.29	83.33	93.4	35.7
HNR	0.289	11.61	0.656	0.563-0.741	58.06	70.83	88.5	30.4
LMR	0.401	3.33	0.745	0.656-0.821	81.72	58.33	88.4	45.2
HMR	0.302	104.9	0.667	0.574-0.752	67.74	62.50	87.5	33.3
TNM	0.979	2	0.993	0.956-1.000	97.85	100.00	100.0	92.3
Joint tests	0.563	0.844	0.846	0.768-0.906	68.82	87.50	95.5	42.0

It can be concluded from the data in **Table 3** that the AUC values for the four combinations of NLR, LMR, HNR, HMR and NLR+LMR+HNR+HMR were higher and had better diagnostic performance. The AUCs of the joint NLR, LMR, HNR, HMR and NLR+LMR+HNR+HMR were compared using Medcalc software, and the differences were not statistically significant (P > 0.05) when the AUCs of the individual tests were compared with each other, and the differences were statistically significant (P < 0.05) when the AUCs of the 4 joint tests were compared with each individual test (**Table 4**).

**Table 4:** The analysis of AUC area of NLR, LMR, HNR, HMR and Joint tests.

Inspection items	Z value	P value
Joint trial with NLR	2.170	0.030
Joint trial with LMR	2.273	0.023
Joint trial with HMR	3.311	<0.001
Joint trial with HNR	3.012	0.003
NLR with LMR	0.090	0.928
NLR with HNR	1.734	0.083
NLR with HMR	0.811	0.417
LMR with HNR	0.951	0.342
LMR with HMR	1.610	0.107
HNR with HMR	0.136	0.892

### Clinical decision curve analysis of NLR, LMR, HNR, HMR and the combination of the four tests in NSCLC metastasis

**Figure 4:**
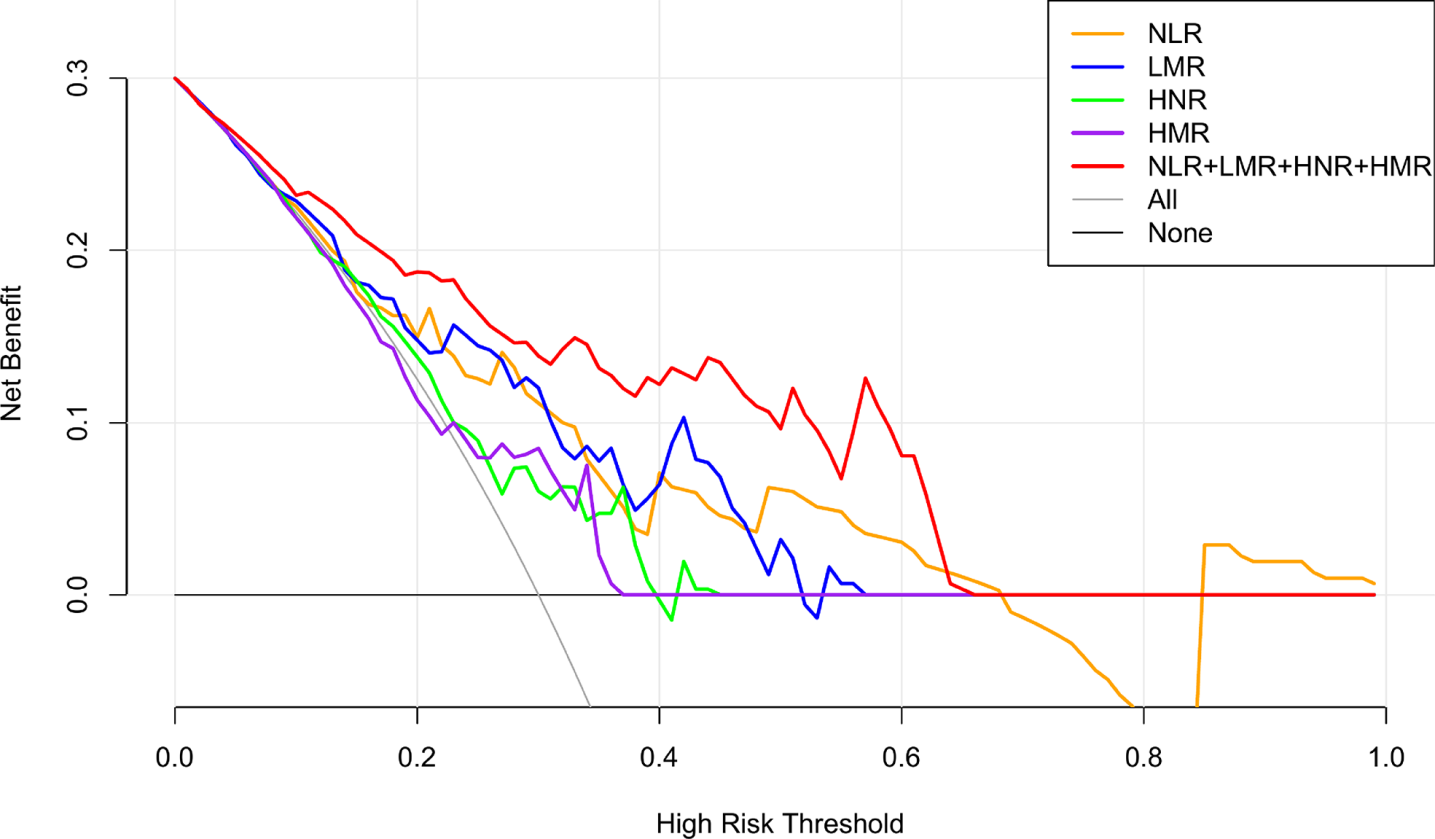
NLR, LMR, HNR, HMR and the clinical decision curve of NSCLC metastasis. (Note: the x-axis represents the threshold probability, the Y-axis represents the net benefit, the black line represents the assumption that all patients did not have metastases, and the gray line represents the assumption that all patients did have metastases).

The net clinical benefit of the model was assessed using the clinical decision curve (DCA), which was determined by the net benefit under different threshold probabilities. The net benefit was determined by subtracting the proportion of false-positive patients from the proportion of true-positive patients and by weighing the relative harms of not intervening and the negative consequences of intervening unnecessarily. The clinical decision curves showed that the net clinical benefit of the prediction models was greater than that of all patients when the threshold probabilities were 9%-68% for NLR alone, 2%-56% for LMR alone, 7%-39% for HNR alone, 18%-36% for HMR alone, and 1%-65% for the threshold probabilities when all four were combined. metastatic or non-metastatic scenarios. The graphical results show that the overall clinical benefit rate of the 4 joint tests was better than that of the single test, as shown in **Figure 4**.

### Risk assessment of NLR, LMR, HNR, HMR in predicting NSCL C metastasis

The risk of NLR, LMR, HNR, and HMR levels in NSCLC metastasis was evaluated using binary logistic regression analysis with cutoff values (two group classification) and quartiles (P25, P50, P75) as cutoff points (four group classification), respectively. The dependent variable y is grouped according to whether metastasis is present (metastasis = 1, no metastasis = 0), and the median and quartile cutoff values of NLR, LMR, HNR, and HMR are grouped as independent variables. The risk value of each index for predicting NSCLC metastasis in the different groups is calculated. In the forest map, the odds ratio (or) is used as an indicator of the effect size of each indicator. In the forest map, the vertical coordinate line takes the estimated value of the effect point = 1 as an invalid line. If the horizontal line segment in the forest map intersects with the vertical coordinate line, it means that there is no statistical difference in risk between groups. If the horizontal line segment in the forest map does not intersect with the vertical coordinate line and lies on the right side of the vertical coordinate line, it means that the risk of the current analysis group is greater than that of the reference group.

**Figure 5:**
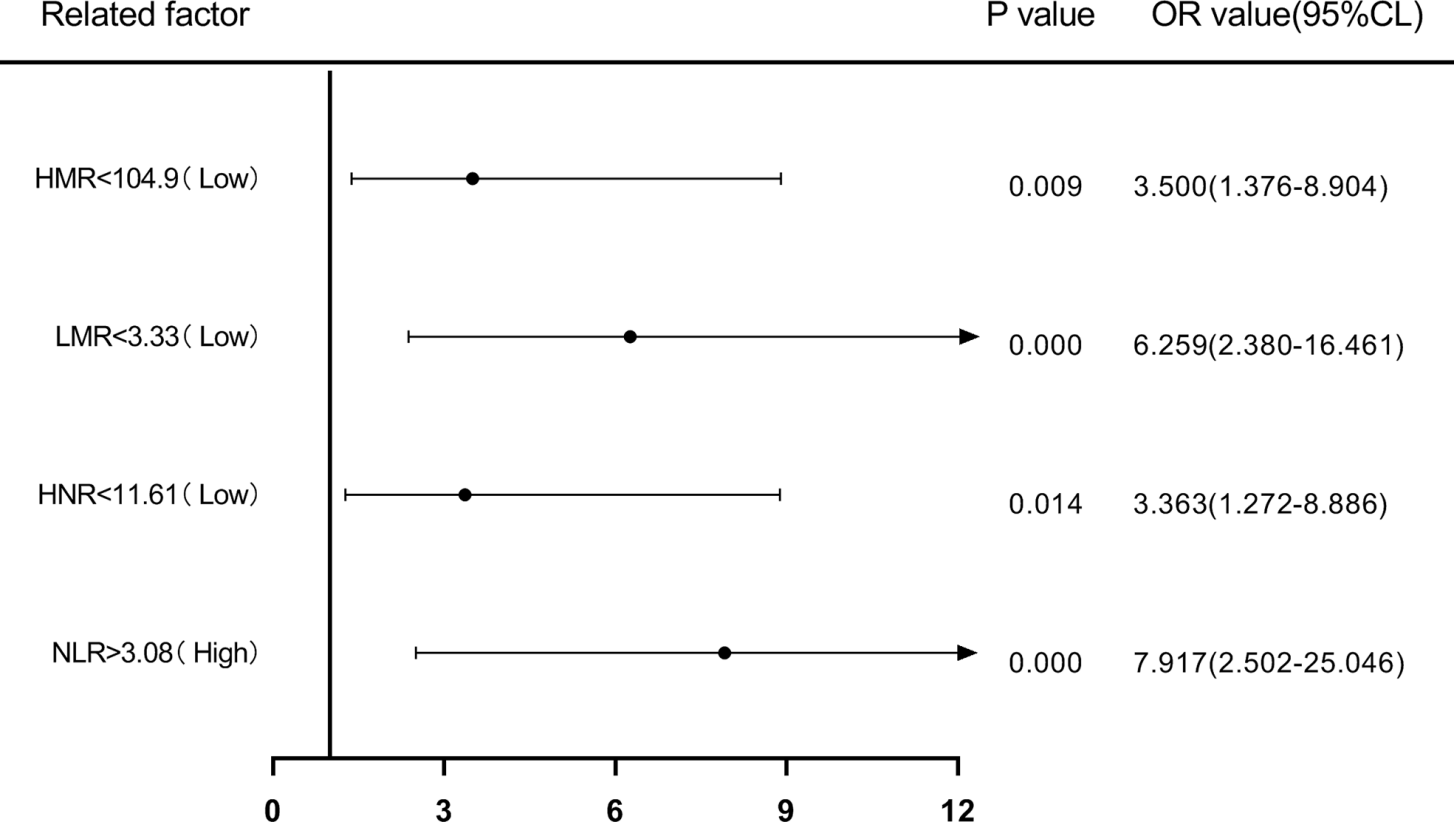
Forest plot of one-way logistic regression analysis of NLR, LMR, HNR, and HMR (two classification groups) in predicting NSCLC metastasis.

**Figure 6:**
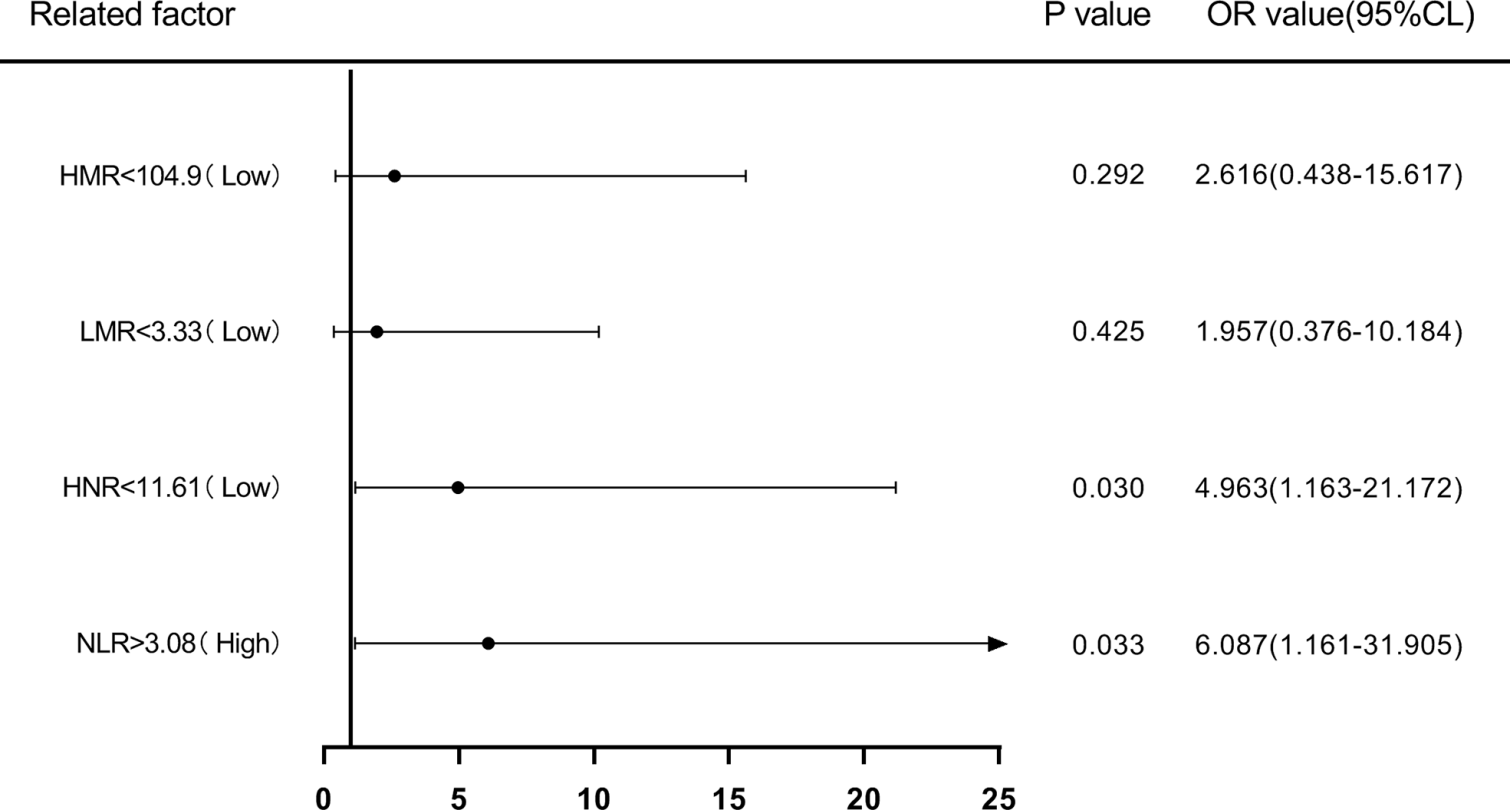
Forest plot of multifactorial logistic regression analysis of NLR, LMR, HNR, and HMR (two groups of classification) in predicting NSCLC metastasis. (Note: multifactor correction included variables ANC, ALC, AMC, HDL).

Patients were first divided into low level and high level groups based on the cutoff values of NLR (3.08), LMR (3.33), HNR (11.61), and HMR (104.9). The OR for the risk of developing NSCLC metastasis was 7.917 (95% CL, 2.502-25.046) (P < 0.05) in patients with high level NLR compared with low level NLR (P < 0.05), while the corrected OR was 6.087 (95% CL, 1.161-31.905) (P < 0.05); compared with high level HNR, patients with low level HNR The OR for the risk of developing NSCLC metastasis was 3.363 (95% CL, 1.272-8.886) compared with high level HNR (P < 0.05), while the corrected OR was 4.963 (95% CL, 1.163-21.172) (P < 0.05); compared with high level LMR, the OR for the risk of developing NSCLC metastasis in patients with low level LMR was 6.259 (95% CL, 2.380-16.461) (P < 0.05), with a concurrent corrected OR of 1.957 (95% CL, 0.376-10.184) (P > 0.05); the OR for the risk of NSCLC metastasis in patients with low-level HMR compared with high-level HMR was 3.500 (95% CL, 1.376- 8.904) (P < 0.05), while the corrected OR was 2.616 (95% CL, 0.438-15.617) (P > 0.05); (**Figures 5**, **6**; **Tables 5**, **6**).

**Table 5:** Univariate Logistic regression analysis of NLR, LMR, HNR, and HMR (two categories) in predicting NSCLC transfer.

Related factors	B	S.E.	Wald	P value	OR value	95%CI	
						lower limit	upper limit
NLR>3.08 (High)	2.069	0.588	12.396	0.000	7.917	2.502	25.046
HNR<11.61 (Low)	1.213	0.496	5.982	0.014	3.363	1.272	8.886
LMR<3.33 (Low)	1.834	0.493	13.818	0.000	6.259	2.380	16.461
HMR<104.9 (Low)	1.253	0.476	6.914	0.009	3.500	1.376	8.904

**Table 6:** Multivariate Logistic regression analysis of N L R, LMR, HNR, and HMR (classified in the two-group categories) in the prediction of NSCLC metastasi.

Related factors	B	S.E.	Wald	P value	OR value	95%CI	
						lower limit	upper limit
NLR>3.08 (High)	1.806	0.845	4.566	0.033	6.087	1.161	31.905
HNR<11.61 (Low)	1.602	0.740	4.684	0.030	4.963	1.163	21.172
LMR<3.33 (Low)	0.671	0.842	0.636	0.425	1.957	0.376	10.184
HMR<104.9 (Low)	0.961	0.912	1.112	0.292	2.616	0.438	15.617

multivariate adjustment included variables ANC, ALC, AMC, HDL.

**Figure 7:**
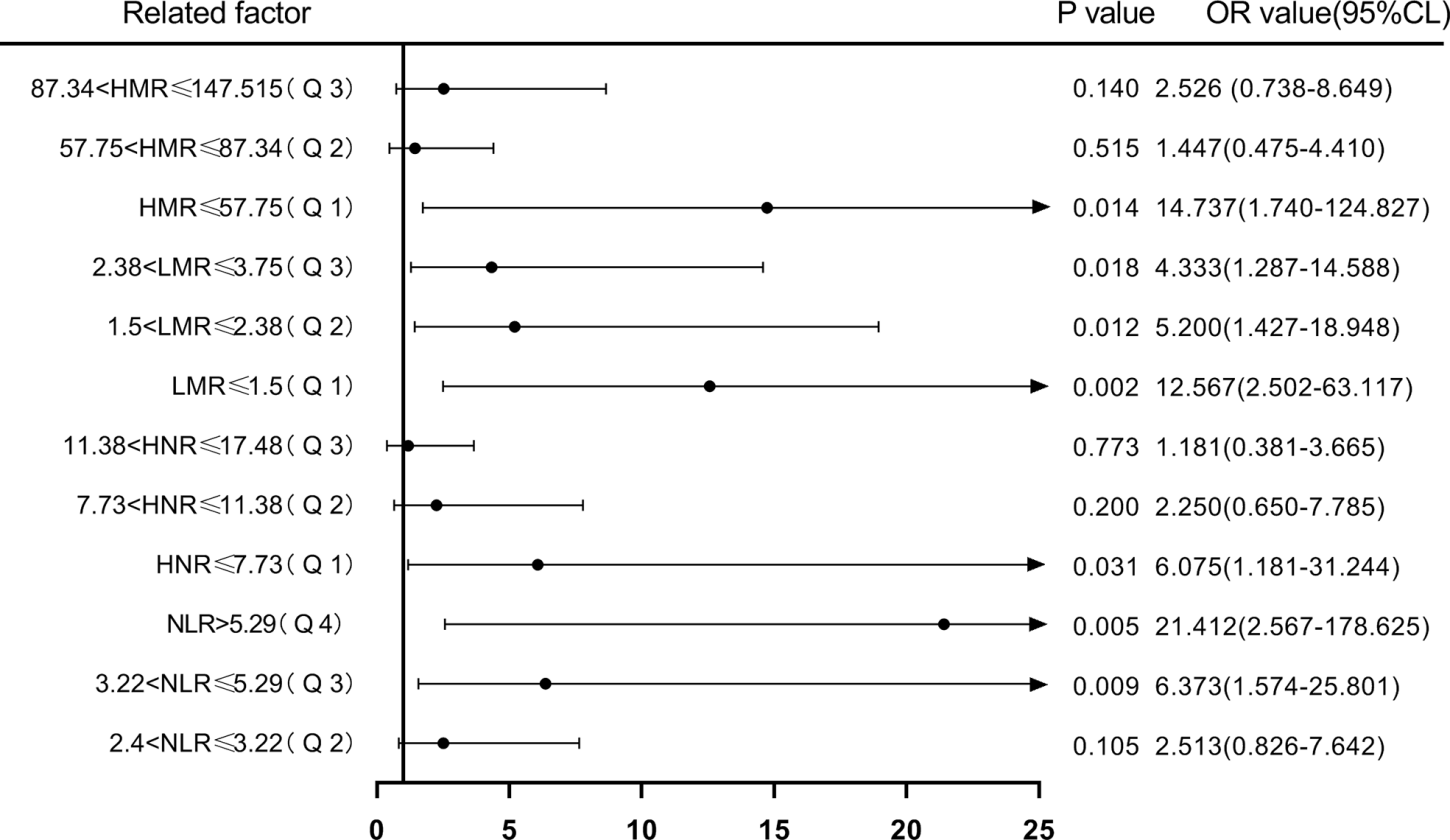
Forest plot of one-way logistic regression analysis of NLR, LMR, HNR, and HMR (four groups of classification) in predicting NSCLC metastasis.

**Figure 8:**
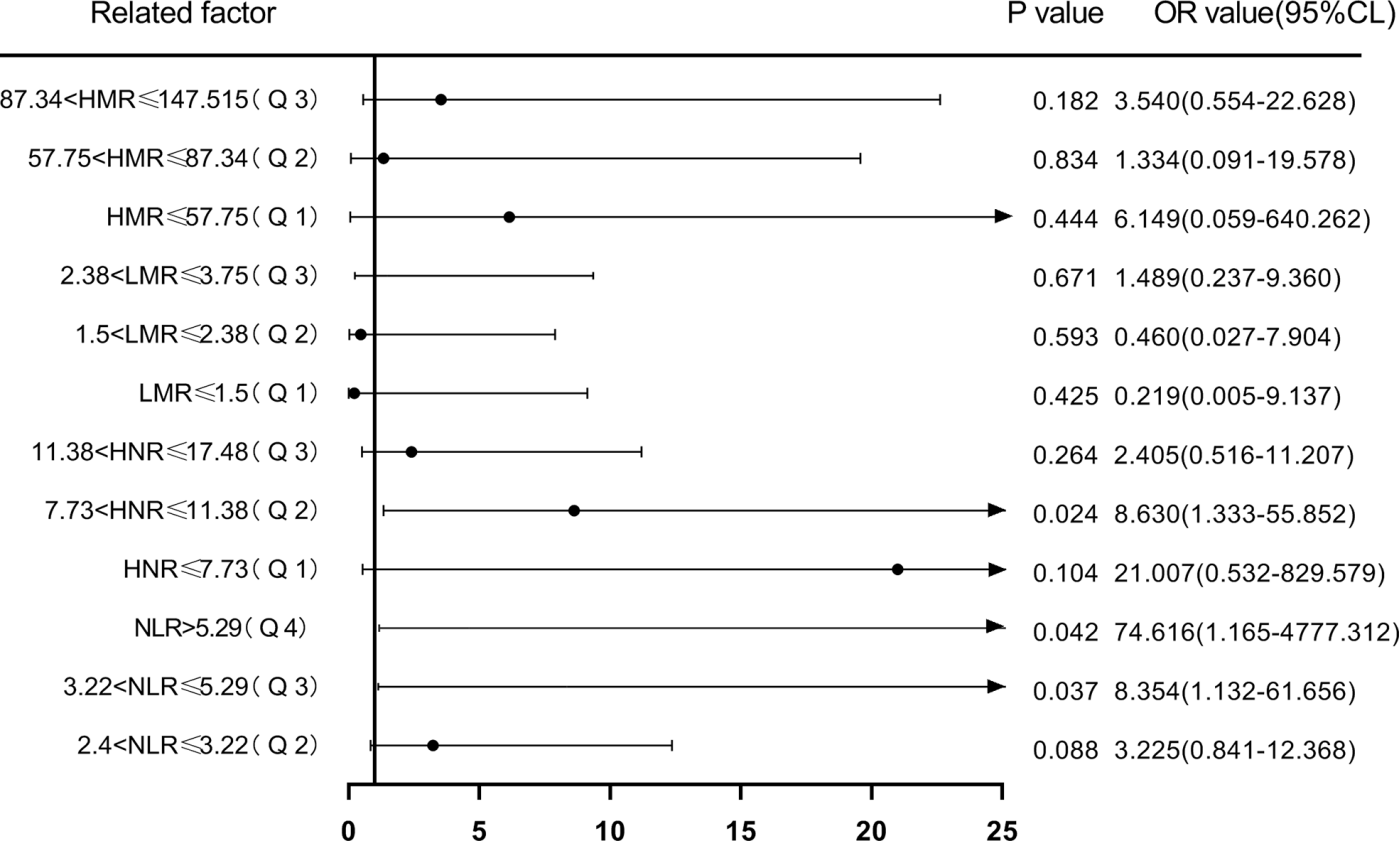
Forest plot of multifactorial logistic regression analysis of NLR, LMR, HNR, and HMR (four groups of classification) in predicting NSCLC metastasis. (Note: multifactor correction included variables ANC, ALC, AMC, HDL).

Next, according to NLR (Q1 ≤ 2.4; 2.4<Q2 ≤ 3.22; 3.22<Q3 ≤ 5.29; Q4>5.29), HNR (Q1 ≤ 7.73; 7.73<Q2 ≤ 11.38; 11.38<Q3 ≤ 17.48; Q4>17.48), LMR (Q1 ≤ 1.5; 1.5<Q2 ≤ 2.38; 2.38<Q3 ≤3.75; Q4 >3.75), HMR (Q1 ≤ 57.75; 57.75<Q2 ≤ 87.34; 87.34<Q3 ≤ 147.515; Q4>147.515) quartiles, and the patients were sequentially divided into Q1, Q2, Q3, and Q4 groups from low to high levels. Compared with the lowest NLR level group (Q1), the ORs for the risk of NSCLC metastasis in the Q2, Q3, and Q4 groups were 2.513 (0.826-7.642), 6.373 (1.574-25.801), and 21.412 (2.567-178.625), respectively, while the corrected ORs were 3.225 (0.841- 12.368), 8.354 (1.132-61.656), and 74.616 (1.165-4777.312), respectively; compared with the highest HNR level group (Q4), the ORs for the risk of NSCLC metastasis in the Q1, Q2, and Q3 groups were 6.075 (1.181-31.244), 2.250 (0.650-7.785), and 1.181 (0.381-3.665), while the corrected ORs were 21.007 (0.532-829.579), 8.63 (1.333-55.852), and 2.405 (0.516-11.207), respectively; compared with the highest LMR level group (Q4), the risk of NSCLC metastasis in the Q1, Q2, and Q3 groups was ORs were 12.567 (2.502-63.117), 5.200 (1.427-18.948), and 4.333 (1.287-14.588), respectively, with concurrent corrected ORs of 0.219 (0.005-9.137), 0.460 (0.027-7.904), and 1.489 (0.237- 9.360); compared with the highest HMR level group (Q4), the ORs for the risk of NSCLC metastasis in the Q1, Q2, and Q3 groups were 14.737 (1.74-124.827), 1.447 (0.475-4.410), and 2.526 (0.738-8.649), respectively, while the corrected ORs were 6.149 (0.059-640.262), 1.334 (0.091-19.578), and 3.540 (0.554-22.628); (**Figures 7**, **8**; **Tables 7**, **8**).

**Table 7:** Univariate Logistic regression analysis of N L R, LMR, HNR, and HMR (all four categories) in the prediction of NSCLC metastasis.

Related factors	B	S.E.	Wald	P value	OR value	95%CI	
						lower limit	upper limit
NLR (Q2)	0.921	0.568	2.635	0.105	2.513	0.826	7.642
NLR (Q3)	1.852	0.713	6.737	0.009	6.373	1.574	25.801
NLR (Q4)	3.064	1.082	8.014	0.005	21.412	2.567	178.625
HNR (Q1)	1.804	0.836	4.662	0.031	6.075	1.181	31.244
HNR (Q2)	0.811	0.633	1.639	0.200	2.250	0.650	7.785
HNR (Q3)	0.167	0.578	0.083	0.773	1.181	0.381	3.665
LMR (Q1)	2.531	0.823	9.448	0.002	12.567	2.502	63.117
LMR (Q2)	1.649	0.660	6.245	0.012	5.200	1.427	18.948
LMR (Q3)	1.466	0.619	5.605	0.018	4.333	1.287	14.588
HMR (Q1)	2.690	1.090	6.091	0.014	14.737	1.740	124.827
HMR (Q2)	0.370	0.568	0.423	0.515	1.447	0.475	4.410
HMR (Q3)	0.927	0.628	2.178	0.140	2.526	0.738	8.649

**Table 8:** Multivariate Logistic regression analysis of N L R, LMR, HNR, H N R and HMR (classified four groups) in the prediction of NSCLC metastasis.

Related factors	B	S.E.	Wald	P value	OR value	95%CI	
						lower limit	upper limit
NLR (Q2)	1.171	0.686	2.914	0.088	3.225	0.841	12.368
NLR (Q3)	2.123	1.020	4.333	0.037	8.354	1.132	61.656
NLR (Q4)	4.312	2.122	4.129	0.042	74.616	1.165	4777.312
HNR (Q1)	3.045	1.876	2.636	0.104	21.007	0.532	829.579
HNR (Q2)	2.155	0.953	5.116	0.024	8.630	1.333	55.852
HNR (Q3)	0.878	0.785	1.249	0.264	2.405	0.516	11.207
LMR (Q1)	-1.517	1.903	0.635	0.425	0.219	0.005	9.137
LMR (Q2)	-0.776	1.451	0.286	0.593	0.460	0.027	7.904
LMR (Q3)	0.398	0.938	0.180	0.671	1.489	0.237	9.360
HMR (Q1)	1.816	2.370	0.587	0.444	6.149	0.059	640.262
HMR (Q2)	0.288	1.371	0.044	0.834	1.334	0.091	19.578
HMR (Q3)	1.264	0.946	1.784	0.182	3.540	0.554	22.628

multivariate adjustment included variables ANC, ALC, AMC, HDL.

## Discussion

Lung cancer is one of the prevalent malignancies worldwide and the leading cause of cancer deaths. Reports show nearly 800,000 new lung cancer cases in China in 2018, with more than 400,000 deaths in men and 200,000 deaths in women, of which NSCLC accounts for the majority of lung cancer (25). With the newer advances in targeted therapy and surgical techniques, the prognosis of NSCLC patients has significantly improved, however, most patients have already developed local or distal metastases at the first visit or follow-up, delaying the optimal treatment window, thus non-invasive biomarkers that can identify the occurrence of metastases in NSCLC are crucial.

In this study, we analyzed the diagnostic value of NLR, LMR, HNR, and HMR in NSCLC metastasis, and the results showed that the level of NLR in the metastatic group of NSCLC was higher than that in the non-metastatic group (P < 0.001), and the levels of LMR, HNR, and HMR in the metastatic group were lower than those in the non-metastatic group (P < 0.001). A comparative analysis of the levels of NLR, LMR, HNR, and HMR in patients with different stages was also performed, and the results showed that NLR levels showed an increasing trend with increasing TNM stages, and LMR, HNR, and HMR levels showed a decreasing trend with increasing TNM stages. The report showed that (26–28) NLR, LMR, HDL and other indicators have a certain correlation with tumor occurrence, development and metastasis, which is basically consistent with this study, indicating that NLR, LMR, HNR and HMR have a certain value in NSCLC metastasis.

Using medcalc software to produce the area under the ROC curve for each index and combined test, the AUC values of NLR, LMR, HNR, HMR and NLR+LMR+HNR+HMR were higher than other indexes, indicating that these four indexes have better diagnostic value in NSCLC metastasis. NLR is a commonly used index calculated from the indexes ANC, ALC, which respond to inflammation. Reports show that (29) elevated NLR can be used as a predictor of tumor types such as breast cancer, gastric cancer, ovarian cancer, pancreatic cancer, etc. The AUC value of LMR test is 0.745, and LMR is an inflammatory index calculated from ALC and AMC. Lymphocytes play an important role in resisting tumors, and decreased lymphocytes will lead to a weakened response of the body to resist tumors, while monocytes, as participants in the inflammatory response (30). The ANC and AMC have a role in promoting tumor progression. The ANC can secrete pro-angiogenic and anti-apoptotic factors, which indirectly provide an environment for tumor cell growth and metastasis (31). HDL plays a role in tumor cell development by suppressing the immune response, and reduced HDL levels predict poor tumor prognosis. Patients with poor prognosis, and for every 10 mg/dl increase in HDL level, cancer incidence is reduced by 36%, and HDL level shows a positive correlation with overall survival of tumor patients (32). By comparing the AUCs of NLR, LMR, HNR, HMR and the 4 joint tests, the AUCs of each single test index were not statistically significant when compared with each other (P > 0.05), and the 4 joint tests were higher than the AUCs of each single test (P < 0.05), indicating that the 4 joint tests were superior to the single test indexes in the diagnostic performance of NSCLC metastasis. The clinical decision curve analysis of NLR, LMR, HNR, HMR and the 4 joint indicators also showed that each single indicator had a better clinical benefit rate in different threshold probability ranges, and the overall clinical benefit rate of the combined indicators was better than that of the single indicators. The overall clinical benefit rate of the combined assay was better than that of the single assay, suggesting that the combined assay of the four clinical indicators can be used as a reference indicator for the disease progression of NSCLC patients.

The risk of NLR, LMR, HNR, and HMR levels in NSCLC metastasis was evaluated using binary logistic regression analysis with cutoff values (two groups of classification) and quartiles (P25, P50, P75) as cut points (four groups of classification), respectively. The results showed that when the cutoff value was used as the cut point, the OR for the risk of NSCLC metastasis was 6.087 (P < 0.05) in patients with high level NLR compared with the low level group after correction, and the OR for the risk of NSCLC metastasis was 3.363 (P < 0.05) in patients with low level HNR compared with high level. When quartiles were used as cut points for four-group classification, the ORs for the risk of NSCLC metastasis in patients in the corrected high level NLR (Q3, Q4) group compared with the low level Q1 group were 8.354 and 74.616, respectively (P < 0.05); the OR for the risk of NSCLC metastasis in patients in the low level HNR Q2 group compared with the high level Q4 group was 8.63 (P < 0.05). The remaining differences were not statistically significant in the low-level LMR and HMR groups compared with the high-level group (P > 0.05). The results suggest that NLR and HNR can be used as risk predictors for the development of metastasis in NSCLC patients, however, LMR and HMR are not sufficient as risk predictors for the development of metastasis in NSCLC patients.

In summary, NLR, LMR, HNR, and HMR have good diagnostic value in NSCLC metastasis. NLR and HNR can be used as risk predictors for metastasis in patients with NSCLC, and NLR, LMR, HNR, and HMR can be easily calculated based on blood cell counts and lipid tests. and the advantage of being easy to perform in primary care institutions. It is worth noting that the present study is a retrospective study with a small sample size, and there may be bias in diagnostic value and unavoidable selection bias. Because of the ratio of follow-up time, the study could not evaluate the long-term prognosis of patients with metastases. More samples will be collected and a prospective study will be designed in a later period to evaluate the long-term metastasis of patients, such as 3–5 years, whether transmission has occurred, to better evaluate the diagnostic value of each index in NSCLC patients with metastasis.

## Data availability statement

The original contributions presented in the study are included in the article. Further inquiries can be directed to the corresponding authors.

## Ethics statement

The study was approved by the Ethics Committee of Shanghai Tongji hospital (2021KYSB061), and the methods were designed according to the Declaration of Helsinki. Because the data were analyzed retrospectively, written informed consent from the subjects was waived.

## Author contributions

WZ: formal analysis, investigation, visualization, writing-original draft. WW: investigation, methodology. JW: investigation, methodology. JT: investigation, methodology. WY: investigation, methodology. YYu: methodology, supervision. YYa: methodology, supervision. AS: resources, project administration. WQ: conceptualization, funding acquisition, methodology, writing-review and editing. All authors contributed to the article and approved the submitted version.

## Funding

This work was supported by the National Natural Science Foundation of China (81902984); the “Gan Quan Xin Xing” talent training program of Shanghai Tongji Hospital (HRBC2005), the Yancheng city medical science and technology development plan project (YK2019112) and the Medical Research Project of Jiangsu Provincial Health and Health Commission (2019179).

## Conflict of interest

The authors declare that the research was conducted in the absence of any commercial or financial relationships that could be construed as a potential conflict of interest.

## Publisher’s note

All claims expressed in this article are solely those of the authors and do not necessarily represent those of their affiliated organizations, or those of the publisher, the editors and the reviewers. Any product that may be evaluated in this article, or claim that may be made by its manufacturer, is not guaranteed or endorsed by the publisher.

## Abbreviations

NSCLC, Non-small cell lung cancer; NLR, Neutrophil/lymphocyte; LMR, lymphocyte/monocyte; HNR, High density lipoprotein/neutrophil; HMR , High density lipoprotein/monocyte; NSE, neuron-specific enolase; Lnc RNA, long-non-coding RNA; ct DNA, circulating tumor DNA; IASLC, International Association for the Study of Lung Cancer; MRI, magnetic resonance imaging; PET-CT, positron emission tomography-computed tomography; NPV, negative predictive value; PPV, positive predictive value; AUC, area under the curve; DCA, decision curves.
