# Corrigendum: Immune cell-lipoprotein imbalance as a marker for early diagnosis of non-small cell lung cancer metastasis

**DOI:** 10.3389/fonc.2022.1118042

**Published:** 2022-12-15

**Authors:** Wei Zhang, Weiwei Wang, Junlu Wu, Jiale Tian, Wenhui Yan, Yi Yuan, Yiwen Yao, Anquan Shang, Wenqiang Quan

**Affiliations:** ^1^ Department of Laboratory Medicine, Shanghai Tongji Hospital, School of Medicine, Tongji University, Shanghai, China; ^2^ Department of Pathology, Tinghu People’s Hospital, Yancheng, China; ^3^ Department of Laboratory Medicine, Yangzhi Rehabilitation Hospital (Shanghai Sunshine Rehabilitation Center), Tongji Univeirsity School of Medicine, Shanghai, China; ^4^ Department of Internal Medicine V-Pulmonology, Allergology, Respiratory Intensive Care Medicine, Saarland University Hospital, Homburg, Germany

**Keywords:** NLR, LMR, HNR, NSCLC, risk assessment, diagnostic markers

In the published article, an author name was incorrectly written as Wenqian Quan. The correct spelling is Wenqiang Quan.

The authors apologize for this error and state that this does not change the scientific conclusions of the article in any way. The original article has been updated.

